# Effect of Chemokine Receptors CCR7 on Disseminated Behavior of Human T cell Lymphoma: clinical and experimental study

**DOI:** 10.1186/1756-9966-30-51

**Published:** 2011-05-07

**Authors:** Jing Yang, Shengyi Wang, Guofan Zhao, Baocun Sun

**Affiliations:** 1 Department of Pathology, Tianjin Medical University, Tianjin, China; 2 Department of Psychology, Tianjin Medical University, Tianjin, China; 3 Department of Pathology and Cancer Hospital of Tianjin Medical University, Tianjin, China

## Abstract

**Background:**

The expression of chemokine receptors CCR7 has been studied in relation to tumor dissemination and poor prognosis in a limited number of cancers. No such studies have been done on CCR7 expression in non-Hodgkin's lymphoma (T-NHL). Our aim in this paper is to investigate the association between CCR7 expression and progression and prognosis of T-NHL.

**Methods:**

1) Analysis of clinical data: The specimens were obtained from 41 patients with T-NHL and 19 patients with lymphoid hyperplasia. Their corresponding clinicopathologic data were also collected. The expression levels of CCR7, MMP-2, and MMP-9 were examined by immunohistochemical staining. 2) Human T-NHL cell lines Hut 78 (cutaneous T-cell lymphoma) and Jurkat (adult T-cell leukemia/lymphoma) were cultured. The invasiveness of the two cell lines were measured with a Transwell invasion assay, and then used to study the effects of chemokine receptors on T-NHL invasion and the underlying molecular mechanism. The transcript and expression of CCR7 were evaluated using RT-PCR and western blotting.

**Results:**

1) The higher CCR7 and MMP-9 expression ratios were significantly associated with multiple lesions and higher stage III/IV. Moreover, a positive correlation was observed between CCR7 and MMP-9 expression. 2) The Hut 78 cell line was more invasive than the Jurkat cells in the Transwell invasion assay. The transcript and expression levels of CCR7 were significantly higher in Hut78 than that of Jurkat cell line. The T-NHL cell lines were co-cultured with chemokine CCL21 which increased the invasiveness of T-NHL cell. The positive association between CCL21 concentration and invasiveness was found. 3) The stronger transcript and expression of PI_3_K, Akt and p- Akt were also observed in Hut78 than in Jurkat cell line.

**Conclusions:**

High CCR7 expression in T-NHL cells is significantly associated with lymphatic and distant dissemination as well as with tumor cell migration and invasion *in vitro*. Its underlying mechanism probably involves the PI_3_K/Akt signal pathway.

## Background

Currently, tumor growth and metastatic dissemination result from a complex, dysregulated molecular machinery, leading to resistance of tumor cells to apoptosis, tumor cell migration, tumor cell invasion, and tumor cell immune escape mechanisms. Recent data suggest that chemokine receptors may direct lymphatic and hematogenous spread, and may additionally influence the sites of metastatic growth of different tumors[1].

Chemokine receptors are GTP-proteins linked to 7 transmembrane domains and they are expressed on the cell membranes of immune and endothelial cells. CCR7, the receptor for chemokine CCL21, was first discovered on B cells infected by Epstein-Barr virus [2]. It is often expressed on naive T cells, memory T cells, B cells, and mature dendritic cells [3,4]. CCR7 is important for lymphatic cell migration and chemotaxis to lymph nodes. CCR7 has two ligands, CCL19 and CCL21. CCL21 and CCR7 are very important for T cell migration, activation, and existence, especially for lymphocytic chemotaxis.

The prominent biological behavior of T-NHL is invasion. Patients often visit doctors when they develop multiple disseminated tumor sites. Normal T cells express CCR7, and when cancer occurs, we have been unable to determine if chemokine receptor expression increase and whether it promoted tumor growth and dissemination. The role of chemokine receptors in tumor spreading has been the focus of recent studies. High CCR7 expression has been associated with lymph node metastases and poor prognosis in oral squamous cell carcinoma and melanoma [5,6]. Supporting data from *in vitro *and murine tumor models underline the key roles of two receptors, CCR7 and CXCR4 in tumor cell malignancy. Stimulation of CCR7 by its ligand CCL21 induces migration and invasion of CCR7-expressing cancer cells [7]. Furthermore, inhibition of the chemokine receptors, such as CXCR4 and SDF-1, could suppress chemokine-induced migration, invasion, and angiogenesis [8,9]. However, no studies have been done on CCR7 expression in human T-NHL and its effects on disease progression and prognosis. Therefore, we evaluated CCR7 expression in T-NHL cell lines and specimens, and analyzed its correlation with clinicopathologic parameters of patients. Our results reveal that high CCR7 expression significantly influences lymphatic and hematogenous tumor dissemination, and also correlates with clinical staging. Moreover, we investigated the underlying mechanisms. We found that high CCR7 expression is associated with lymphatic and distant dissemination in patients with T-NHL, probably via the PI3K/Akt signal pathway.

## Methods

### Clinical Data

#### Materials

We collected 41 specimens of T-cell non-Hodgkin's lymphoma and 19 lymph nodes of reactive hyperplasia from 2003 to 2008 in the General Hospital of Tianjin Medical University. All specimens were formalin-fixed and embedded in paraffin. Not all patients underwent treatment on their visits. Of the 41 T-NHL patients, 23 were males and 18 were females. The mean age was 48.34 ± 16.19 years. According to the WHO classification, the histological types of the specimens in our study included peripheral T cell lymphoma, not otherwise characterized (32 cases), extranodal NK/T cell lymphoma, nasal type (5 cases), anaplastic large cell lymphoma (2 cases), and angioimmunoblastic T cell lymphoma (2 cases).

#### Method

##### Immunohistochemical Staining

The avidin-biotin complex method was used to detect the CCR7 (anti-CCR7, 1:300 dilution; Epitomics Inc.), MMP-2 (anti-MMP-2, 1:250 dilution; Zhong Shan Inc., Beijing), and MMP-9 (anti-MMP-9, 1:250 dilution; Zhong Shan Inc., Beijing). The formalin-fixed, paraffin-embedded tissues were deparaffinized and subsequently heated in a microwave oven with EDTA buffer. After preincubation with hydrogen peroxide, an avidin/biotin blocking kit, and rabbit serum, the primary antibodies were applied overnight in the wet box at 4°C, and then incubated with the secondary antibodies (rabbit anti-goat biotinylated; 1:200 dilution, ZhongShan Inc., Beijing) for about 50min. At last avidin-biotin complex was added, and enzyme activity was visualized with diaminobenzidine. Counterstaining was done with hematoxylin. For the negative controls, only the secondary antibodies were used. A negative control was done for every lymphoma and reactive lymph node sample (n = 60). For the positive controls, formalin-fixed, paraffin-embedded tissue samples of the human spleen were applied.

##### Evaluation of Immunohistochemical Staining

Immunohistochemical staining was independently evaluated by four authors, blinded to patient outcome and all clinicopathologic findings. The immunohistochemical staining was analyzed according to staining index, which was calculated by multiplying the score for staining intensity (0, absent, no color in tumor cells; 1, weak, pale yellow in tumor cells; 2, intermediate, yellow in tumor cells; 3, strong staining, brown yellow in tumor cells) with the score for percentage of stained tumor cells (0, positive cells account for 0%-10%; 1, 11%-25%; 2, 26%-50%; 3, >50%). The staining index value ranges from 0 to 9. The specimens grouped by staining index value as - (<2), + (2-4), ++ (5-7), +++ (8-9). The slide of ++ or higher than ++ was classified as high expression. Otherwise, the slide was classified as low expression. The slides were usually evaluated by four observers. The final classification of a slide was determined by the value agreed to by a majority of observers.

### In vitro Experimentation

#### Materials

##### Cell Culture

The human cutaneous T cell lymphoma cell line Hut78 and the adult T lymphocytic leukemia/lymphoma Jurkat cell line were inoculated into cellular culture boards with improved 1640 medium supplemented with 10% fetal bovine serum (Hyclone, Inc., USA), 100 units/mL penicillin, 100 μg/mL streptomycin (Cambrex, East Rutherford, NJ), and 1 mmol/L L-glutamine. CCL21 were mixed into media to final concentrations of 50 (S_50 _group), 100 (S_100 _group), and 200 nmol/L (S_200 _group). Two cell lines were aggregated and grown in the same suspension.

#### Method

##### RNA Isolation and Semiquantitative Reverse Transcriptase Polymerase Chain Reaction (RT-PCR)

RNA isolation was done using the RNeasy Kit according to the manufacturer's recommendations (Biomiga Inc., American). Gene transcriptions of actin, CCR7, PI3K, and Akt were analyzed via a two-step RT-PCR. Reverse transcription was done with 2 μg of RNA (20 μL total volume; Omniscript RT Kit, Qiagen) according to the manufacturer's recommendations. Up to 1 μL of cDNA was used as a template for the specific PCR reactions. The primers used were as follows: β-actin, forward *5'-CCTGGGCATGGAGTCCTGTG-3' *and reverse *5'-AGGGGCCGGACTCGTCATAC-3' *(305 bp fragment); CCR7, forward *5'-TCCTTCTCATCAGCAAGCTGTC-3' *and reverse *5'-GAGGCAGCCCAGGTCCTTGAA-3' *(529 bp fragment); PI3K, forward *5'-CATCACTTCCTCCTGCTCTAT-3' *and reverse *5'-CAGTTGTTGGCAATCTTCTTC-3' *(377 bp fragment); Akt, forward *5'-GGACAACCGCCATCCAGACT-3' *and reverse *5'-GCCAGGGACACCTCCATCTC-3' *(121 bp fragment). For amplification, a DNA Engine PTC200 (MJ Research, Watertown, MA) thermocycler was used. The cycling conditions for the respective PCRs were as follows: initial denaturation (10 min at 95 °C) followed by 35 cycles of denaturation (30 s at 94 °C), annealing (30 s at the following temperatures: β-actin, 59 °C; CCR7, 53 °C; PI3K, 53 °C; Akt, 56 °C), and elongation (1 min at 72 °C). After the last cycle, a final extension (10 min at 72 °C) was added and, thereafter, the samples were kept at 4 °C. Then, 5 μL of the products were run on a 1% agarose gel under a constant voltage of 100 V for 20 min, stained with ethidium bromide, and then analyzed it under UV light.

##### Western Blot Analysis

Hut 78 and Jurkat cells were washed in PBS and lysed in RIPA lysate solution (Solarbio Inc.). Then, 100 μg of protein were separated by 10% SDS-PAGE. After separation, the protein were transferred from the gel onto a polyvinylidene difluoride membrane. The respective proteins were detected by anti-CCR7 (1:3000, Epitomics Inc., 1:1000 rabbit anti-goat IgG second antibodies, Zhongshan Inc., Beijing), anti-Akt (1:1000, Beyotime Inc., Shanghai, 1:1000 rabbit anti-goat IgG second antibodies, Zhongshan Inc., Beijing), anti-p-Akt (1:2000, Epitomics Inc., 1:1000 rabbit anti-goat IgG second antibody, Zhongshan Inc., Beijing), and anti-GAPDH (1:1000, Santa Cruz, America; 1:1000 goat anti-rabbit IgG second antibodies, ZhongShan Inc., Beijing), and were visualized with an ECL Western blotting analysis system.

##### Cellular Invasion Assays

Invasiveness assays of Hut 78 and Jurkat cells were performed in a Transwell chamber. (8 μm pore size; Corning Inc.). Each group of cells was centrifuged and washed in PBS, resuspended with supernatant, and adjusted to a cellular density of 5 × 10^5^. Then, 100 μL of the cell suspension from each group was placed into the upper Transwell chambers and 600 μL of culture fluid with the corresponding CCL21 concentration was placed into the lower chamber. The chambers were then incubated for 24 hours at 37 °C in a humid atmosphere of 5% CO_2_. After incubation, the number of cells that migrated to the lower chamber was determined with eosin staining. The cells entered the substrate in the lower chamber and then were mixed uniformly. At last, we counted the cells under the microscope (10 randomly selected high power fields) individually.

### Statistical Analysis

Data were analyzed with SPSS 11.5 software. Statistics processing about clinical data were evaluated with *χ*^2 ^test, Spearman's rank correlation test. Statistics processing about *in vitro *experimentation were t test and ANOVA. P < 0.05 was considered significant and P < 0.001 highly significant in all statistical analyses.

## Results

### Immunohistochemical Staining of CCR7, MMP-9, and MMP-2 (Table 1)

**Table 1:** The chemokine receptor expression ratios of T-NHL group and comparison group [number of cases (%)]

Group	n	CCR7	MMP-9	MMP-2
T-NHL group	41	34 (82.9)	36 (87.8)	29 (70.7)
Control group	19	3 (15.8)	3 (15.8)	2 (10.5)
*χ*^2^		32.219*	29.598*	18.845*

*P < 0.01

**Figure 1:**
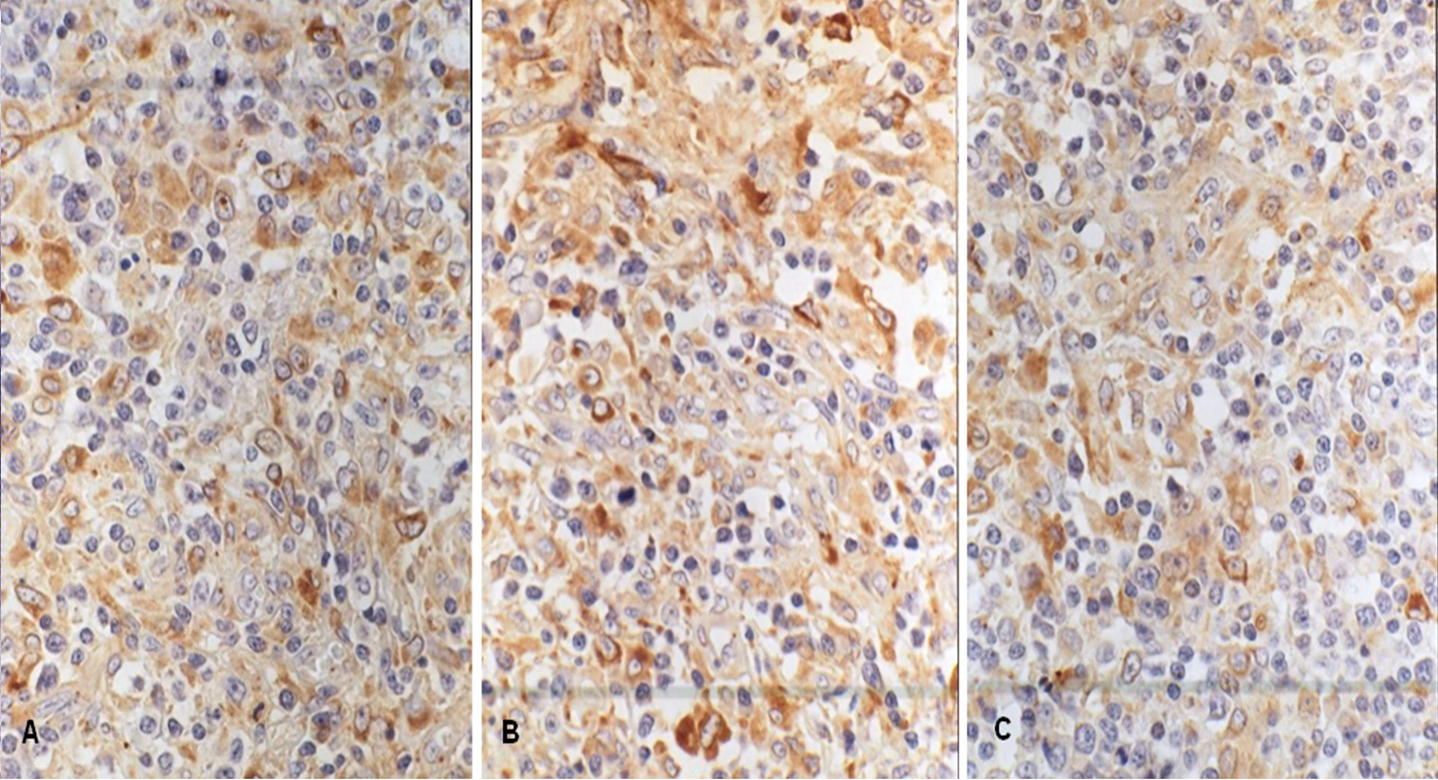
**The expression of CCR7, MMP-9 and MMP-2 in T-NHL with immunohistochemical staining**. These markers all express in the cytoplasm. Some yellow or brown yellow granules in the cytoplasm are postive. The immunohistochemical staining was performed with S-P method and these photoes were taken under the high power (×400). A was CCR7 stainting. The staining intensity is strong. B was MMP-9 stainting. The staining intensity is strong. C was MMP-2 staining. The staining intensity is intermediate.

The result for CCR7, MMP-9, and MMP-2 revealed a predominantly cytoplasmic staining. A focal weak membrane staining (Figure 1) was observed. The high expression ratio of CCR7, MMP-9, and MMP-2 were 82.9%, 87.8%, and 70.7% in T-NHL specimens, respectively. All markers' high expression ratios were higher than that in hyperplastic lymph node group (P < 0.01).

### Expression of all parameters in T-NHL group and correlation with clinical parameters

(1) There was no significant correlation of high CCR7 expression ratio with age (87.5% >60 years vs 81.8% <=60 years), sex (87% males vs. 77.8% females) and tumor size (88.0% >3 cm vs. 75.0% <3 cm) (Table 2). The positive correlation between high CCR7 expression and multiple location dissemination was found. The CCR7 expression ratio of the multiple locations group was higher than that in the single location group (92.6% vs. 64.3%, *P *< 0.05). Concerning WHO classification, the high expression ratio of CCR7 also was highly significantly associated with higher tumor UIUC stages. UICC stage III and IV group had 100% high CCR7 expression compared with 75% in UICC stage I and II group(*P *< 0.05).

**Table 2:** The correlation between clinical parameters and higher expression of the three pathological parameters [number of cases (%)]

Clinical-Pathology	n	CCR7	MMP-9	MMP-2
Sex				
Male	23	20 (87.0)	20 (87.0)	18 (78.3)
Female	18	14 (77.8)	16 (88.9)	11 (61.1)

Age				
≤60 years	33	27 (81.8)	29 (87.9)	25 (75.8)
>60 years	8	7 (87.5)	7 (87.9)	4 (50)

Tumor size				
≤3 cm	16	12 (75.0)	12 (75.0) *	13 (81.3)
>3 cm	25	22 (88.0)	24 (96.0) *	16 (64)

Clinical Stage				
Stage I-II	24	18 (75.0) *	19 (79.2) *	20 (83.3) *
Stage III-IV	17	17 (100.0) *	17 (100.0) *	9 (52.9) *

B symptom				
No	16	13 (81.3)	13 (81.3)	11 (68.8)
Yes	25	21 (84.0)	23 (92.0)	18 (72)

Location				
Single location	14	9 (64.3) *	10 (71.4) *	12 (85.7) *
Multiple location	27	25 (92.6) *	26 (96.3) *	17 (63) *

* *P *< 0.05

(2) The MMP-9 expression ratio in the multiple locations group (96.3%) was higher than that in the single location group (71.4%), in the clinical stage III-IV group (100%) than that in the clinical stage I-II group (79.2%), and in the >3 cm tumor size group that in the ≤3 cm group (96% vs. 75%, *P *< 0.05). MMP-9 expression ratio showed no signification difference in gender and age. The highly positive correlations of MMP-9 expression ratio with multiple location dissemination, higher UICC stages and larger tumor size were observed. (Table 2);

(3) Contrary to CCR7 and MMP-9, MMP-2 showed higher expression in single location group compared with multiple locations group (52.9% vs. 83.3%, *P *< 0.05). MMP-2 expression was also significantly associated with lower UIUC stages (83.3% vs 52.9%).

(4) Other clinical parameters without statistical significance were not included in the table.

### Correlation among all indices in T-NHL

The high expression of CCR7, MMP-9, and MMP-2 in T-NHL was analyzed with Spearman's correlation analysis. The relationship between CCR7 and MMP-9 (rs = 0.395, *P *< 0.05) expressed direct correlation. The relationship among other markers showed no significant correlation (*P *> 0.05).

### Transwell invasion experiment result (Table 3)

**Table 3:** Cellular count in the lower chamber in Transwell invasion experiment ( ± s, n = 9)

	Control group	S_50 _group	S_100 _group	S_200 _group
Jurkat	10.63 ± 5.52	20.70 ± 8.40^✩^	33.43 ± 10.61^✩^	49.13 ± 21.01^✩^
Hut 78	15.00 ± 6.48^⋆^	35.37 ± 18.21^⋆▴^	42.26 ± 20.17^▴^	72.60 ± 34.12^⋆▵^

^⋆^Compared with corresponding group of Jurkat cells, *P *< 0.01;

^✩^Compared with the other groups of Jurkat cells (including the control group), *P *< 0.01;

^▴^Compared with the control group and of S_200 _group of Hut 78 cells, *P *< 0.01;

^▵^Compared with the other groups of Hut 78 cells (including the control group), *P *< 0.01.

In the lower chamber, there were more Hut 78 cells than Jurkat cells in all groups except S_100 _group (*P *< 0.01).

The number of Hut 78 and Jurkat cells that penetrated the membrane in the S_50_, S_100_, and S_200 _groups were all higher than that in the control group (*P *< 0.01).

For the Hut 78 cell line, the cells in the S_200 _group were higher than that in the S_50 _group, whereas for the Jurkat cell line, the cells in the S_100 _group were higher than that in S_50 _group, and the cells in S_200 _were higher than that in S100 group (P < 0.01).

### The expression and transcript of CCR7 in two cell lines under conventional culture and CCL21 co-culture

#### (1) CCR7mRNA transcript (Table 4, Figure 2)

**Table 4:** The relative grey scale of CCR7mRNA transcript ( ± s, n = 9)

	Control group	S_50 _group	S_100 _group	S_200 _group
Jurkat	0.1512 ± 0.0278	0.4604 ± 0.0331^✩^	0.7453 ± 0.0636^✩^	0.9071 ± 0.4985^✩^
Hut 78	0.5282 ± 0.0537^⋆^	0.6943 ± 0.0365^⋆▵^	0.8477 ± 0.0513^⋆▴^	0.8710 ± 0.0485^▴^

^⋆^Compared with the corresponding group of Jurkat cells, *P *< 0.01;

^✩^Compared with the other groups of Jurkat cells (including the control group), *P *< 0.01;

^▴^Compared with the control group and S50 group of Hut 78 cells, *P *< 0.01;

^▵^Compared with the other groups of Hut 78 cells (including the control group), *P *< 0.01.

**Figure 2:**
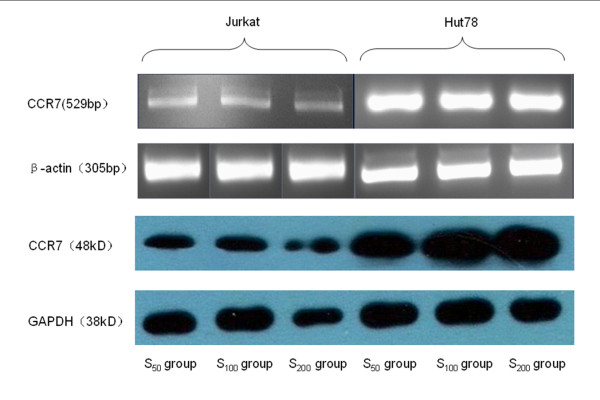
**The expression of CCR7 mRNA and protein in Jurkat and Hut cells after CCL21 co-culture in vitro**. RT-PCR amplication and Western Blot analysis of the two cell lines under the different concentration of CCL21, which was performed as described in Methods. β-actin is positive control in RT-PCR amplication and GAPDH is positive control in Western Blot analysis. The relative grey scale of CCR7 mRNA and protein in Hut cell were both higher than that in Jurkat cell with corresponding concentration of CCL21. In the group with different concentration of CCL21 of each cell lines, there were some differences on the grey scale as described in the result.

According to the relative grey scale, the numbers of CCR7 transcripts of the two cell lines in all concentration groups were higher than that in the control group (*P *< 0.01).

The CCR7 transcripts of the Hut 78 cells in control, S_50, _and S_100 _groups were higher than that in the corresponding groups of Jurkat cells (*P *< 0.01).

The CCR7 transcripts of the two cell lines in the higher concentration group were higher than that in the lower concentration group, except for S_100 _and S_200 _groups in the Hut 78 cell line (*P *< 0.01).

#### (2) Expression of CCR7 protein (Table 5, Figure 2)

**Table 5:** The relative grey scale of CCR7 protein ( ± s, n = 9)

	Control group	S_50 _group	S_100 _group	S_200 _group
Jurkat	0.5053 ± 0.0336	0.4870 ± 0.0278	0.6916 ± 0.0238^✩^	0.7095 ± 0.0332^✩^
Hut 78	1.1037 ± 0.1135^⋆^	1.0700 ± 0.1121^⋆^	1.4792 ± 0.2500^⋆▴^	1.4804 ± 0.2524^⋆▴^

^⋆^Compared with the corresponding group of Jurkat cells, *P *< 0.01;

^✩^Compared with the control group and the S_50 _group of Jurkat cells, *P *< 0.01;

^▴^Compared with the control group and the S_50 _group of Hut 78 cells, *P *< 0.01.

In both cell lines, the relative expression of the CCR7 protein in the S_100 _and S_200 _groups were higher than that in the control group, whereas the CCR7 expression in the S_100 _group was higher than that in the S_50 _group (*P *< 0.01).

The CCR7 expression of the Hut 78 cell line in the control, S_50_, S_100_, and S_200 _groups were higher than those of the Jurkat cell line (*P *< 0.01).

### The expression and activation of PI_3_K/Akt pathway in the two cell lines under conventional culture and CCL21 co-culture

#### (1) PI_3_K mRNA transcript (Table 6, Figure 3)

**Table 6:** The relative grey scale of PI_3_KmRNA ( ± s, n = 9)

	Control group	S_50 _group	S_100 _group	S_200 _group
Jurkat	0.2170 ± 0.0289	0.7897 ± 0.0549^✩^	0.8310 ± 0.0377^✩▵^	0.8248 ± 0.0381^▵^
Hut 78	0.6061 ± 0.0545^#^	0.7996 ± 0.0200^▴^	0.8365 ± 0.0346^▴^	0.8759 ± 0.0467^⋆▴^*

^⋆^Compared with the corresponding group of Jurkat cells, *P *< 0.05;

^# ^Compared with the corresponding group of Jurkat cells, *P *< 0.01;

^✩^Compared with the control group of the Jurkat cells, *P *< 0.01;

^▵^Compared with the other groups of Jurkat cells, including the control group, *P *< 0.05;

^▴^Compared with the control group of Hut 78 cells, *P *< 0.01;

* Compared with the S_50 _group of Hut 78 cells, *P *< 0.01.

**Figure 3:**
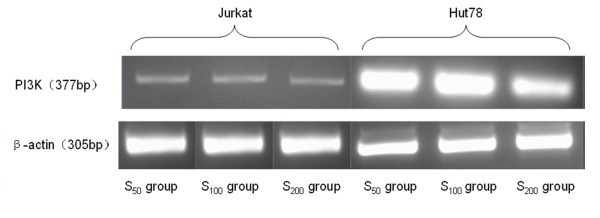
**The expression of PI3K mRNA in Jurkat and Hut cells after CCL21 co-culture in vitro**. RT-PCR amplication of the two cell lines under the different concentration of CCL21. The relative grey scale of PI3K mRNA in Hut cell was higher than that in Jurkat cell with corresponding concentration of CCL21. there were some difference on the grey scale in the group with different concentration of CCL21 of each cell lines. β-actin is positive control in RT-PCR amplication.

The relative PI_3_K mRNA expression levels in all concentration groups were higher than that in the control group (*P *< 0.01). The relative PI_3_K mRNA expression levels of the Jurkat cells in the S_100 _and S_200 _groups were both higher than that in the S_50 _group. The expression in the S_200 _group was lower than that in the S_100 _group (*P *< 0.05). For the Hut 78 cells, there were no significant differences in relative expression levels in all three concentration groups. The relative expression levels in the control and S_200 _groups were both higher than that in the Jurkat cells. The relative expression levels had no significant differences between Hut 78 and Jurkat cells in S_50 _and S_100 _groups.

#### (2) Akt mRNA transcript (Table 7, Figure 4)

**Table 7:** The relative grey scale of the Akt mRNA ( ± s, n = 9)

	Control group	S_50 _group	S_100 _group	S_200 _group
Jurkat	0.1808 ± 0.0264	0.3224 ± 0.0172^✩^	0.5194 ± 0.0340^✩^	0.6305 ± 0.0212^✩^
Hut 78	0.2279 ± 0.0183^⋆^	0.6418 ± 0.0344^⋆▵^	0.7107 ± 0.0149^⋆▵^	0.7325 ± 0.0234^⋆▵^

^⋆^Compared with the corresponding group of Jurkat cells, *P *< 0.01;

^✩^Compared with the other groups of Jurkat cells, including the control group, *P *< 0.01;

^▵^Compared with the other groups of Hut 78 cells, including the control group, *P *< 0.05.

**Figure 4:**
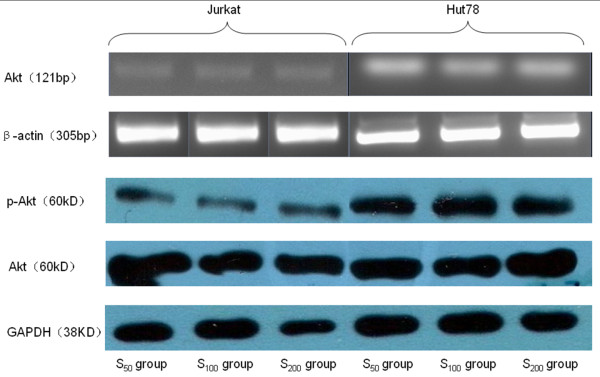
**The expression of Akt mRNA, Akt protein and p-Akt protein in Jurkat and Hut cells after CCL21 co-culture in vitro**. RT-PCR amplication and Western Blot analysis of the two cell lines under the different concentration of CCL21. β-actin is positive control in RT-PCR amplication and GAPDH is positive control in Western Blot analysis. The relative grey scale of Akt mRNA, Akt protein and p-Akt protein in Hut cell were all higher than that in Jurkat cell with corresponding concentration of CCL21.

The relative Akt mRNA expression levels in all concentration groups were higher than that in the control group (*P *< 0.01). The relative Akt mRNA expression levels of the Hut 78 cells in the control, S_50_, S_100_, and S_200 _groups were all higher than those of the Jurkat cells (*P *< 0.05). The relative expression levels of the two cell lines in the higher concentration group were significantly higher than that in lower concentration group (Table 7).

#### (3) p-Akt protein expression (Table 8, Figure 4)

**Table 8:** The relative grey scale of p-Akt protein after co-culture ( ± s, n = 9)

	Control group	S_50 _group	S_100 _group	S_200 _group
Jurkat	0.5523 ± 0.0112	0.5680 ± 0.0566^▵^	0.7784 ± 0.0694^✩^	0.9184 ± 0.0668^✩^
Hut 78	0.9171 ± 0.0483^⋆^	1.1717 ± 0.1679^⋆^*	1.3055 ± 0.0799^⋆▴^	1.1507 ± 0.1010^⋆^*

^⋆^Compared with the corresponding group of Jurkat cells, *P *< 0.01;

^✩^Compared with the other groups of Jurkat cells, including the control group, *P *< 0.01;

^▵^Compared with the S_100_and the S_200 _groups of Jurkat cells, *P *< 0.01;

^▴^Compared with the other groups of Hut 78 cells, including the control group, *P *< 0.01;

* Compared with the control and the S_100 _groups of Hut 78 cells, *P *< 0.01.

For the Hut 78 cells, the relative p-Akt protein expression levels in all concentration groups were all significantly higher than that in control group. The expression in the S_100 _group was significantly higher than those in the S_50 _and S_200 _groups.

For the Jurkat cells, the relative p-Akt protein expression levels of in the S_100 _and S_200 _groups were significantly higher than that in the control group and the expression in the higher concentration group was significantly higher than that in the lower concentration group.

The relative expression levels of Hut 78 cells in the control, S_50_, S_100_, and S_200 _groups were higher than those of Jurkat cells.

## Discussion

This is the first study analyzing the expression profiles of CCR7 chemokine receptors in a larger series of human T cell lymphoma tissues and cell lines. We further determined whether CCR7 expression influenced tumor cell migration *in vitro *and the metastatic behavior of T-NHL and its prognosis in patients, as recently reported for many other malignant tumors. In 2001, Müller [10] first reported breast carcinoma with higher expression of a CCR7 chemokine receptor in primary and metastatic foci. He also found high expression of CCL21 in metastatic sites, such as lymph node, lung, liver, and bone marrow. In an *in vitro *experiment, he found that SDF-1 increased F actin expression in the tumor cells, which can form pseudopodia. In addition, CCL21 also induced breast carcinoma cell migration and basement membrane invasion. CCR7 expression has previously been associated with intrapleural dissemination in non-small cell lung cancer [11], gastric carcinoma [12], and so on, implying the relevant function of CCR7 expressing during carcinogenesis in these cancers. The theory of a CCR7-co-mediated mechanism of lymphatic dissemination was also supported by an animal study, revealing that the CCR7 expression of melanoma cells increases metastases formation in the regional lymph nodes of mice [13]. Moreover, using monoclonal antibodies against CCL21 could prevent lymph node metastasis. CCR7-mediated lymphatic dissemination had been compared with the chemotaxis of activated dendritic cells to CCL21-expressing lymph nodes via lymphatic vessels [7,12,14-16].

Diverse functional studies investigating the influence of CCR7 expression and the activation by its ligand CCL21 were recently conducted, revealing that CCR7 is crucial for adhesion, migration, and invasion of CCR7-expressing malignant tumors [11-13]. To confirm the function of CCR7 in T-NHL, we performed migration and invasion assays using Hut 78 and Jurkat cells. In the vitro experiment, we found that the invasiveness of Hut 78 cell through a Transwell chamber was higher than that of Jurkat cells. Moreover, the CCR7 mRNA transcript and protein expression of Hut 78 cells were also higher than that of Jurkat cells. The migration of these two CCR7 expressing cell lines was significantly stimulated by CCL21, implying an important role and intact function of CCR7 during tumor progression. The invasion capability of these two cell lines is associated with the CCL21 concentration gradient. However, CCR7 protein expression was no significant difference between S_100 _group and S_200 _group. CCR7 expression in S_200 _group was even lower than that in S_100 _group. Therefore, the ideal CCL21 concentration for CCR7 expression in T cell lymphoma is 50-100 nmol/L. This result is consistent to that in the experiment by Mafei [17]. They proposed that the ideal CCL21 concentration for CCR7 expression in breast carcinoma is 50-500 nmol/L. Under this CCL21 concentration, CCR7 can achieve maximum expression in regulating neoplastic cell chemotaxis and invasion. The concentrations beyond 50-500 nmol/L could affect CCR7 expression and subsequently influence chemotaxis and invasiveness. These results indicate that the intensity of CCL21-induced cell migration and invasion *in vivo *correlates with cellular CCR7 expression.

Previous publications have reported that CCR7 activation is critical for metastasis to lymph nodes, lungs, and liver. The mechanism is similar to that of lymphocytic chemotaxis. One study reported that T-cell acute lymphoblastic leukemia is at an increased risk of central nervous system (CNS) relapse. They identified a single chemokine-receptor (CCR7 and CCL19) interaction as a CNS "entry signal" [18]. CCL21 is mainly distributed among peripheral immune organs, especially lymph nodes and spleen. Gunn's study showed that CCL21 could be found in the high endothelial vein of lymph nodes and Peyer's patches, T lymphatic zones, lymphoid follicles, and endothelial cells of lymphatic vessel in many organs. CCL21 can drive lymphocytes in human T cell line and peripheral blood, but not chemotaxis for neutrophils and monocytes, which suggest that CCL21 is specific for the trafficking of T lymphocytes [16]. CCL21 has dual effects on malignant tumor formation. CCL21 can attract immune cells and inhibit vascularization, which block tumor growth. Meanwhile, the increase of CCR7 chemokine receptor expression promotes tumor growth and metastasis. When the latter effect is prominent, the tumor disseminates. Under normal conditions, CCR7 is expressed on T cells. When malignancy occurs, the neoplastic T cell may enhance the expression of CCR7. The differential expression of CCL21 by endothelial cells might explain at least one part of this process. Our results support the chemotaxis theory that CCL21 expression co-mediates the dissemination of primary tumors to different organs [19]. Hasegawa [20] found that adult T cell leukemia/lymphoma (ATLL) cells with high CCR7 expression have increased directional migration capability toward CCL21, which suggests that CCR7 expression may facilitate ATLL cell movement to the high endothelial vein of lymph nodes with abundant CCL21, and then to metastasis.

The influence of CCL21 on lymphatic dissemination (compared with hematogenous) has not been investigated thus far, but CCL21 is also highly expressed in lymph nodes, and CCR7 inhibition results in suppression of breast cancer lymph node metastases, which implies similar pathways for lymphatic and hematogenous dissemination [10].

PI_3_K/Akt, an intracellular signal pathway, plays a role in the invasion of many malignant tumors. Whether PI_3_K/Akt participates in the invasion and metastasis of T cell lymphomas induced by CCR7 and if a relationship exists between them remains unclear.

The PI_3_K/Akt signal pathway was first found in the 1990's. The catalysate of PI3K can participate in cellular proliferation, living, differentiation, and migration [21]. Receptor protein tyrosine kinase (RPTK) activation results in PI(3,4,5)P(3) and PI(3,4)P(2) production by PI3K at the inner side of the plasma membrane. Akt interacts with these phospholipids, causing its translocation to the inner membrane, where it is phosphorylated and activated by PDK1 and PDK2. The activated Akt modulates the function of numerous substrates which are involved in the regulation of cell survival, cell cycle progression, and cellular growth.

Several studies have proven that Akt expression is excessively upregulated in many malignant tumors, such as thyroid carcinomas, gliomas, breast carcinomas, pulmonary carcinomas, and so on [22-26]. As a protein kinase, Akt is activated through phosphorylation. The upregulation of Akt protein may promote oncogenesis and tumor growth. The expression level of phosphorylated-Akt is the indicator of the kinase activity.

In our experiment, the expression levels of PI3K mRNA, Akt mRNA, and p-Akt protein in Hut 78 cells were higher than that in Jurkat cells. The Hut 78 cells were more invasive than the Jurkat cells. The invasiveness of T-NHL is associated with the CCR7 expression. CCR7 is a transmembrane receptor of GTP-protein. CCR7 may activate Akt and the PI3K/Akt signal pathway to promote cell proliferation and spread. Noelia [27] reported that CCR7 could activate the intracellular PI3K/Akt signal pathway to promote cell proliferation and suppress apoptosis in DC cells.

We have a hypothesis about how do CCR7 trigger PI_3_K/Akt signal pathway. The expression of lymph node chemokine in T-NHL could cause the upregulation of chemokine receptors. The interaction between chemokines and their receptors may then activate the Akt protein by peroxodiphosphoric acid, followed by the activation of the PI_3_K/Akt signal pathway, which can promote tumor cell proliferation and invasion. This result provides a theoretical foundation for the targeting of CCR7 and the PI_3_K/Akt signal pathway with antibodies for the treatment of T-NHL. However, further studies on the concrete mechanism of activation of this pathway and its downstream genes are still needed.

In this study, we also detected expression of MMP-9 and MMP-2. MMP is a matrix metalloproteinase that breaks down and destroy Type IV and Type V collagen, as well as gelatin in the extracellular matrix, and then promote tumor metastasis. CCR7 expression in T-NHL was directly correlated with MMP9 expression. High MMP-9 expression has previously been reported in non-Hodgkin's lymphoma [28,29], which can influence the biological behavior and clinical progression of tumor. For T-NHL, a report in an animal experiment found that the high expression of MMP-9 is correlated with liver metastasis [30]. The high expression of MMP-9 is also associated with bad prognosis. The relationship between CCR7 and MMP-9 suggests that these two factors may enhance each other and promote tumor dissemination synergistically. However, the function of MMP-2 in T-NHL metastasis is still unclear.

## Conclusions

Higher CCR7 expression in T-NHL cells is significantly associated with lymphatic and distant dissemination in patients, as well as with migratory and invasive phenotypes *in vitro*. Our study suggested that CCR7 plays an important role in the progression of T-NHL. The possible mechanism is via the PI_3_K/Akt signal pathway. Further studies are needed to evaluate the inhibition of metastatic growth through blocking CCR7 and PI_3_K/Akt signal pathway.

## Competing interests

The authors declare that they have no competing interests.

## Authors' contributions

JY participated in the design of the study, and performed the statistical analysis and drafted the manuscript. She also carried out the cellular culture and RT-PCR assay and western blotting analysis. SYW collected clinical data and carried out immunohistochemistry staining and molecular genetic studies. She also helped to perform the statistical analysis. GFZ participated in clinical data collection and carried out the cellular invasion assay. BCS acquired the funding. He also conceived of the study, and participated in its design, and supervised experimental work and helped to draft the manuscript. All authors read and approved the final manuscript.
